# Accuracy of Individuals Post-hemiparetic Stroke in Matching Torques Between Arms Depends on the Arm Referenced

**DOI:** 10.3389/fneur.2019.00921

**Published:** 2019-08-22

**Authors:** Netta Gurari, Nina A. van der Helm, Justin M. Drogos, Julius P. A. Dewald

**Affiliations:** ^1^Department of Physical Therapy and Human Movement Sciences, Northwestern University, Chicago, IL, United States; ^2^Department of Biomechanical Engineering, Delft University of Technology, Delft, Netherlands; ^3^Department of Biomedical Engineering, Northwestern University, Chicago, IL, United States

**Keywords:** perception, torque, stroke, evaluation methodology, mechatronics

## Abstract

**Background:** Prior work indicates that 50–75% of individuals post-hemiparetic stroke have upper-extremity weakness and, in turn, inaccurately judge the relative torques that their arms generate during a bimanual task. Recent findings also reveal that these individuals judge the relative torques their arms generate differently depending on whether they reference their paretic vs. non-paretic arm.

**Objective:** Our goal was to determine whether individuals with hemiparetic stroke inaccurately matched torques between arms, regardless of the arm that they referenced.

**Methods:** Fifteen participants with hemiparetic stroke and 10 right-hand dominant controls matched torques between arms. Participants performed this task with their right arm referencing their left arm, and vice versa. Participants generated (1) 5 Nm and (2) 25% of their reference elbow's maximum voluntary torque (MVT) in flexion and extension using their reference arm while receiving audiovisual feedback. Then, participants matched the reference torque using their opposite arm without receiving feedback on their matching performance.

**Results:** Participants with stroke had greater magnitudes of error in matching torques than controls when referencing their paretic arm (*p* < 0.050), yet not when referencing their non-paretic arm (*p* > 0.050). The mean magnitude of error when participants with stroke referenced their paretic and non-paretic arm and controls referenced their dominant and non-dominant arm to generate 5 Nm in flexion was 9.4, 2.6, 4.2, and 2.5 Nm, respectively, and in extension was 5.3, 2.8, 2.5, and 2.3 Nm, respectively. However, when the torques generated at each arm were normalized by the corresponding MVT, no differences were found in matching errors regardless of the arm participants referenced (*p* > 0.050).

**Conclusions:** Results demonstrate the importance of the arm referenced, i.e., paretic vs. non-paretic, on how accurately individuals post-hemiparetic stroke judge their torques during a bimanual task. Results also indicate that individuals with hemiparetic stroke judge torques primarily based on their perceived effort. Finally, findings support the notion that training individuals post-hemiparetic stroke to accurately perceive their self-generated torques, with a focus of their non-paretic arm in relation to their paretic arm, may lead to an improved ability to perform bimanual activities of daily living.

## 1. Introduction

Inaccurately judging the relative torques that one generates with their arm(s) can make it difficult to perform bimanual activities of daily living, such as carrying a tray, pushing a grocery cart, and caring for an infant (1, 2). An estimated 500,000 individuals who survive a hemiparetic stroke each year in the USA inaccurately judge the relative torques that they generate between arms (3–11). Even so, a gap remains in our understanding of the extent to which these erroneous judgments occur. Our goal in this work was to determine whether individuals with hemiparetic stroke inaccurately match torques between arms when referencing each arm while flexing and extending about their elbow.

Assessments of one's ability to accurately judge their self-generated torques often request an individual to generate symmetric torques between their limbs without receiving feedback on their matching performance (4, 5, 7–19). Based on findings from such assessments, the current understanding is that individuals perceive their self-generated torques by combining peripheral and central information (1, 15, 20–26). Peripheral information arises from muscle and cutaneous mechanoreceptors (22, 27). Muscle mechanoreceptors include Golgi tendon organs (28–31) and muscle spindles (32, 33), and cutaneous mechanoreceptors include slow-adapting and fast-adapting units (34–37). Central information arises from an individual's perceived effort, or the extent to which an individual perceives that their arm muscles are being driven in relation to the maximum. Findings from ongoing research indicate that the greater an individual's strength asymmetry, the poorer their ability to accurately perceive torques between limbs (4, 7–11, 13–19, 38, 39). In turn, the general understanding is that: (i) perceived effort information generated in the cortex is weighed more heavily than sensory information arising from the periphery (2) and (ii) matching errors occur because individuals do not adapt their perceived effort at their affected limb to its weakened state. Findings from the literature have also provided controversial results as to whether arm dominance affects an individual's judgment of their self-generated forces (10, 12, 16, 19, 40–44).

We know that, post-stroke, portions of an individual's motor pathways (e.g., corticospinal tracts) may be damaged (45), leading to reduced motor unit rate modulation and mean firing rates (46), as well as muscular atrophy (47). In turn, individuals post-hemiparetic stroke may generate a maximal effort at their paretic arm that results in a lower torque output than a maximal effort at their non-paretic arm. Researchers have suggested that individuals with hemiparetic stroke are unable to adapt their perceived effort in their paretic limb to its weakened state and, in turn, do not generate symmetric torques in the absence of visual and corrective feedback (4, 5, 7–11). Our preliminary results, however, challenge this understanding by suggesting that the ability to accurately judge self-generated torques may depend on whether individuals with hemiparetic stroke reference their paretic vs. non-paretic arm (10). Moreover, Yen and Li (11) reported that the accuracy with which torques are matched between arms changes depending on the arm that individuals reference.

In this work, we assessed an individual's ability to accurately judge their self-generated torques between arms when referencing each arm. We ran this assessment during elbow flexion and extension tasks so that we could determine the extent to which impairments in individuals with stroke depend on the direction of the applied force. We compared the accuracy of individuals with stroke in matching torques between arms to individuals without neurological impairments (i.e., controls) so that we could gain an improved understanding of the degree to which their judgment may be erroneous. By using a mechatronic system to execute our assessment, we could control the user's interaction through automated audiovisual cues, such that the delivery of the assessment was highly repeatable. Additionally, we could quantify the user's ability to accurately match torques between arms to a high degree of resolution, such that the outcome measures were objective and highly sensitive. Based on our preliminary work in this area (10), we hypothesized that individuals with hemiparetic stroke would match torques between arms with a similar accuracy as controls if they referenced their non-paretic arm, yet inaccurately if they referenced their paretic arm. This asymmetry in their ability to accurately match torques between arms would support our hypothesis that individuals with hemiparetic stroke do not learn post-injury how to perceive their non-paretic arm in relation to their paretic arm.

## 2. Methods

We refer the reader to van der Helm et al. (10) for a more in-depth description of the experimental methods.

### 2.1. Participants

This study was approved by the Northwestern University Institutional Review Board (STU00021840), and all participants provided written informed consent. Non-Northwestern University employees were monetarily compensated. Participants were required to: have the capacity to provide informed consent; understand and be able to execute the task; and not have any serious upper-extremity or peripheral nerve injury that may interfere with their torque generation/perception. Participants were assessed for their arm dominance using the Edinburgh Handedness Inventory (48).

Participants with stroke were screened by a licensed physical therapist (co-author Dr. Justin Drogos, PT/DPT/NCS) for potentially relevant impairments. This therapist assessed these participants for motor and sensory impairments using the upper-extremity Fugl-Meyer Motor Assessment (UE FMA) (49) and revised Nottingham Sensory Assessment (rNSA) (50), respectively. In addition to the recruitment criteria listed above, participants with stroke were required to have: a single unilateral brain lesion greater than 6 months prior to testing; lesion(s) located above the brainstem, yet not in the cerebellum; not used antispastic agents (e.g., baclofen) in the past 6 months that may impact their task performance; and no comorbid neurological impairments. Lesion location(s) was determined from medical records and T1/T2 MRI scans.

### 2.2. Experimental Setup

**Figure 1:**
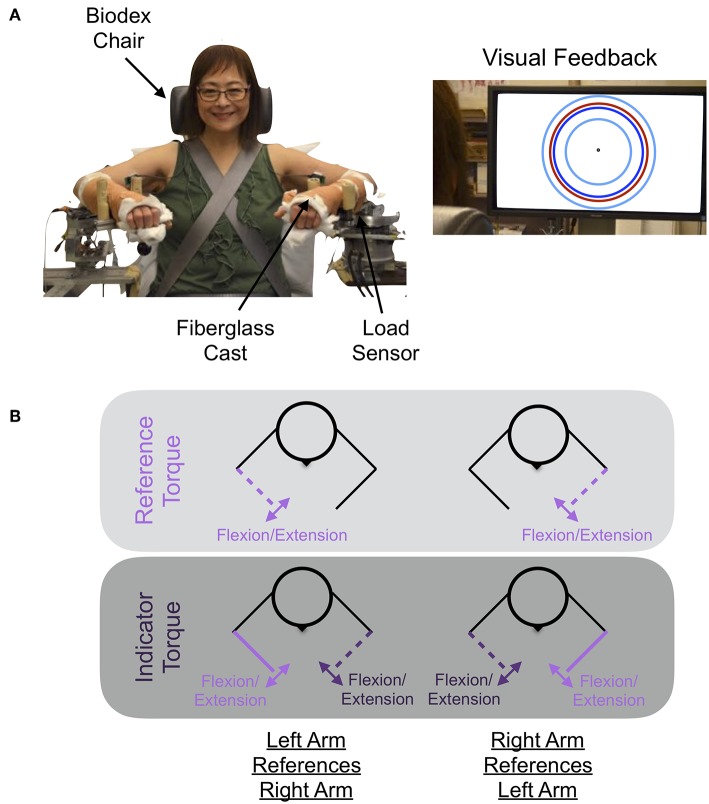
Experimental setup and protocol. **(A)** Shown is the mechatronic system that was used to assess each participant's accuracy and precision in matching torques between arms when referencing each arm. The participant received visual feedback on the monitor about their reference arm's applied torque (red circle), the target torque (dark blue circle), and the allowable range of applied torques (cyan inner and outer circles). **(B)** The participant generated a reference torque in flexion or extension with their reference arm (light purple) while receiving visual feedback, and then matched this torque using their opposite, i.e., indicator, arm (dark purple). Written informed consent was obtained for the publication of the image. Images are adapted from van der Helm et al. (10) ©IEEE.

The experimental setup used to quantify how accurately and precisely participants matched torques between arms is shown in Figure 1. The participant's self-generated elbow torque was measured at each arm using a one-degree-of-freedom load sensor (OMEGA Engineering Inc. LCM201-300; Stamford, CT, USA). A monitor provided the participant with a visual indicator of the magnitude of their self-generated torque at their reference arm, and speakers provided the participant with auditory cues indicating the actions to execute with each arm throughout the duration of each trial. Trial-related data were stored at 1 kHz.

### 2.3. Torque-Matching Trial

The participant was instructed to match, between arms, sub-maximal isometric torques that they generated about their elbow joints as accurately as possible. Automated audio cues were played to encourage that the events occurring throughout a trial were consistent across trials and participants.

**Figure 2:**
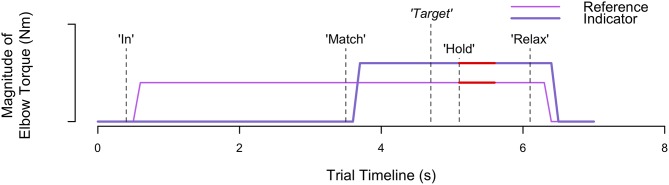
Trial timeline. Visually depicted are the torques generated and corresponding events occurring throughout an example trial when each participant matched torques between arms. The automated audio cues are indicated by the vertical dashed lines. Participant verbal feedback, indicating when the torques are matched, is indicated by the vertical dashed line with the italicized word “*Target.”* The red thick horizontal lines identify the 0.5 s of data extracted to calculate the reference and indicator torque that the participant generated.

Figure 2 visually depicts the torques that the participant generated at each arm throughout a trial, along with the corresponding automated audio cues. At the start of each trial, an automated auditory cue, “In” or “Out,” instructed the participant to flex or extend, respectively, their reference forearm about the elbow joint. The monitor visually portrayed the target torque, τ_target_, using a dark blue circle. The participant generated a torque with their reference arm to reach the target torque, and their applied torque was visually conveyed using a red circle. Once the participant maintained for 3 consecutive seconds their applied torque within an acceptable range of torques ($0.8\cdot \tau_{\text{target}}<{\hat{\tau }}_{\text{user}}<1.2\cdot \tau_{\text{target}}$), as visually depicted using two cyan circles, an automated auditory cue, “Match,” instructed the participant to use their opposite indicator arm to match the reference torque. No feedback was provided about the torque applied by the indicator arm. Once the participant perceived that the torque generated by each arm was the same, the participant stated aloud “*Target.”* At this time, the experimenter pressed a designated keyboard key to indicate that the torques were matched. Following, an automated auditory cue, “Hold,” instructed the participant to maintain the same torque in each arm. After 1 s, the automated auditory cue, “Relax,” instructed the participant to relax both arms, marking completion of the trial. No feedback was provided to the participant about their accuracy in matching torques between arms.

### 2.4. Experimental Protocol

The participant was instructed to not exercise the day prior to and of each testing session to avoid fatigue. Each testing session was conducted on a separate day.

During the first testing session, the maximum voluntary torque (MVT) that the participant could generate about the elbow joint of each arm was quantified in flexion and extension. During the second testing session, the accuracy and precision with which the participant could match sub-maximal isometric torques between arms when referencing each arm was evaluated. The procedures followed for each session are provided in more detail in van der Helm et al. (10); we outline the latter procedures here.

At the beginning of the second testing session, the participant's body was secured to a Biodex chair (System 3 Pro™; Shirley, NY, USA), using straps, such that trunk and shoulder movements were restricted. The participant's forearms were casted to: (i) permit a rigid connection between their isometric movements and recordings from the isometric measurement devices and (ii) encourage that, when applying torques to each isometric device, the participant was cutaneously stimulated evenly across the length of each forearm. The participant was situated in each isometric measurement device with shoulder abduction angles of 85°, shoulder flexion adduction angles of 40°, and elbow flexion angles of 90°. The 90° elbow flexion angle corresponds to the approximate configuration at which the MVT about the elbow joint can be generated (51).

Prior to initiating the testing trials, we determined whether the participant could generate and hold each arm within the desired range of torques. The participant was required to flex and extend each arm to 20 and 40% of their paretic arm's MVT in flexion and extension, and to continue holding the desired torque within the required range of torques (i.e., $0.8\cdot \tau_{\text{target}}<{\hat{\tau }}_{\text{user}}<1.2\cdot \tau_{\text{target}}$) for 3 consecutive seconds. As such, we could determine whether the participant's motor impairments would interfere with their ability to match torques on our task.

To quantify the accuracy and precision with which torques were matched between arms, the participant completed eight consecutive testing trials for each of the eight testing conditions (i.e., 2 reference arms × 2 directions × 2 target torques). Thus, the participant completed eight blocks of eight testing trials, where each block was comprised of a single testing condition. The participant rested for a minimum of 20 s between every trial to permit their involuntary muscle activity (i.e., hypertonicity) to subside (52). Presentation order of the eight testing conditions was randomized across participants using a latin square design.

A target torque of 5 Nm (i.e., τ_target_ = 5) was included in the testing to identify participant perception during a situation that resembles lifting an object that has a fixed mass. We refer to this task as a fixed torque task. A target torque of 25% of MVT (i.e., τ_target_ = 0.25 · τ_MVT : ref_) was included to account for the fact that generating 5 Nm of elbow torque corresponds to a different degree of challenge across participants, due to variations in their strength. We refer to this task as a percentage of MVT task.

### 2.5. Data Analyses

#### 2.5.1. Strength Asymmetry

We quantified the asymmetry in participant strength between arms for each direction (i.e., flexion, extension) using the MVTRatio, which we defined as the MVT of the paretic arm, τ_MVT : par_, divided by the MVT of the non-paretic arm, τ_MVT : nonPar_, in participants with stroke and the MVT of the non-dominant arm, τ_MVT : nonDom_, divided by the MVT of the dominant arm, τ_MVT : dom_, in controls. An MVTRatio of 1.0 indicates that the strength is identical in each arm. An MVTRatio less than 1.0 indicates that the paretic arm in participants with stroke and non-dominant arm in controls is weaker than the non-paretic arm in participants with stroke and dominant arm in controls, respectively. An MVTRatio greater than 1.0 indicates that the paretic arm in participants with stroke and non-dominant arm in controls is stronger than the non-paretic arm in participants with stroke and dominant arm in controls, respectively.

#### 2.5.2. Torque-Matching Ability

We assessed the accuracy and precision with which each participant matched sub-maximal isometric torques between arms for each of the eight testing conditions (i.e., 2 reference arms×2 directions×2 target torques). First, we identified for every trial, *i*, the indicator torque, ${\hat{\tau }}_{\text{ind},i}$, and reference torque, ${\hat{\tau }}_{\text{ref},i}$. These torques were defined as the average of 0.5 s of torque data from the indicator arm and reference arm, respectively, immediately after the experimenter pressed a keyboard key in response to the participant stating that the torques were matched (see Figure 2 for a visual depiction). Next, we identified the torque-matching error, τ_err, *i*_, for every trial, *i*, or the difference between the indicator torque, ${\hat{\tau }}_{\text{ind},i}$, and reference torque, ${\hat{\tau }}_{\text{ref},i}$. A negative and positive torque-matching error corresponded to the participant undershooting and overshooting the reference torque, respectively. We visually inspected participant torque-matching errors as a function of trial, for each testing condition, to verify that learning, fatigue, or boredom, as indicated by trends of increasing or decreasing errors, did not occur.

Following, we identified participant accuracy using the constant error and absolute error, and participant precision using the variable error (10, 53, 54). Constant error, absolute error, and variable error were determined for each of the eight testing conditions (i.e., 2 reference arms×2 directions×2 target torques) based on the eight testing trials of every condition. Constant error, CE, is the mean torque-matching error across the eight testing trials and identifies whether the participant consistently underestimated (CE < 0) or overestimated (CE > 0) the reference torque. Absolute error, AE, is the mean magnitude of the torque-matching error across the eight testing trials and identifies whether the participant consistently perfectly matched (AE = 0) or poorly matched (AE >> 0) the reference torque. Variable error, VE, is the standard deviation of the torque-matching error across the eight testing trials and identifies whether the participant matched using consistently the same torque (VE = 0) or highly variable torques (VE >> 0).

We also wanted to address whether torques were matched between arms based on the effort that the participant perceived was required to activate each limb rather than the magnitude of the torques that they generated. As such, we normalized the torque about each elbow by the corresponding MVT and identified the normalized torque-matching error, τ_norm, err, *i*_, as a percentage, e.g.,:

(1) $$\begin{array}{r} \tau_{\text{norm},\text{err},i}=100\times \left(\frac{{\hat{\tau }}_{\text{ind},i}}{{\hat{\tau }}_{\text{MVT},\text{ind}}}-\frac{{\hat{\tau }}_{\text{ref},i}}{{\hat{\tau }}_{\text{MVT},\text{ref}}}\right). \end{array}$$

We determined the mean normalized error across the eight testing trials for each condition (i.e., 2 reference arms×2 directions×2 target torques).

### 2.6. Statistical Analyses

We determined whether accuracy and precision in matching torques differed depending on the arm participants referenced (i.e., controls: dominant/non-dominant; participants with stroke: paretic/non-paretic) for each tested direction and target torque. This analysis was achieved using linear-mixed effects models (55, 56), where reference arm was a fixed effect and participant was a random effect. Outcome measures were CE, AE, and VE, as well as the mean normalized error. For models with a significant main effect, we identified significantly differing levels (57), using a Holm correction to account for the multiple comparisons (58). Mean (μ), standard deviation (σ), and standard error (SE) values are reported.

## 3. Results

Data were collected from fifteen participants with hemiparetic stroke and ten participants without neurological impairments (i.e., controls). Tables 1, 2 provide demographic, clinical, and experimental information about each participant.

**Table 1:** Participant clinical and experimental information.

**Participant**	**UE**	**rNSA elbow**	**rNSA elbow**	**Lesion**	**τ_*MVT*:*flex*_**	**τ_*MVT*:*flex*_**	**τ_*MVT*:*ext*_**	**τ_*MVT*:*ext*_**
	**FMA**	**kinaesthesia**	**pressure**	**location(s)**	**Par/Non-dom**	**Non-Par/dom**	**Par/Non-dom**	**Non-par/dom**
	**score**	**sensation**	**sensation**	**(L: Left/R: Right)**	**arm (Nm)**	**arm (Nm)**	**arm (Nm)**	**arm (Nm)**
		**score**	**score**					
Stroke 1	48	2	2	L: IC	87.5	90.7	57.3	70.6
Stroke 2	26	2	2	NA	53.5	85.9	43.5	64.8
Stroke 3	39	–	–	L: Th, IC, BG, F, P	51.6	90.4	41.4	62.4
Stroke 4	62	3	2	L: F, P	37.2	43.8	24.9	20.6
Stroke 5	38	2	2	R: Th, IC	40.9	68.4	26.3	46.5
Stroke 6	24	2	2	R: Th, IC, BG	37.9	69.0	15.0	59.0
Stroke 7	18	2	1	R: Th, IC, BG	14.1	77.9	31.8	66.8
Stroke 8	29	2	1	R: BG, F, I	32.2	51.9	22.6	29.9
Stroke 9	57	2	2	L: IC, BG	60.5	72.3	49.6	45.6
Stroke 10	49	2	2	R: Th, IC, I	28.2	39.9	23.9	37.8
Stroke 11	58	3	2	L: T-P	41.7	48.1	30.5	31.1
Stroke 12	12	2	2	R: IC, F, P, O	14.7	63.9	18.1	55.1
Stroke 13	26	0	0	L: Th, IC, BG	17.6	50.6	22.0	48.0
Stroke 14	25	NA	NA	L: IC, BG	18.5	25.8	18.8	19.0
Stroke 15	39	3	2	R: IC	56.3	58.3	39.7	51.1
Control 1	–	–	–	–	34.0	36.7	24.5	25.8
Control 2	–	–	–	–	76.9	74.8	55.0	55.1
Control 3	–	–	–	–	34.1	33.6	18.5	20.1
Control 4	–	–	–	–	111.3	119.3	73.7	77.5
Control 5	–	–	–	–	31.5	35.7	18.6	21.5
Control 6	–	–	–	–	40.8	44.1	25.6	28.8
Control 7	–	–	–	–	46.3	44.0	35.3	36.8
Control 8	–	–	–	–	45.0	51.1	35.1	36.5
Control 9	–	–	–	–	40.6	41.4	26.3	24.5
Control 10	–	–	–	–	71.0	72.8	48.2	50.9

*UE FMA, upper-extremity Fugl-Meyer Motor Assessment; rNSA, revised Nottingham Sensory Assessment; τ_MVT : flex_, elbow flexion maximum voluntary torque for every participant with stroke (paretic, non-paretic) and control (dominant, non-dominant); τ_MVT : ext_, elbow extension maximum voluntary torque for every participant with stroke (paretic, non-paretic) and control (dominant, non-dominant); Dom, dominant; Non-Dom, non-dominant; Par, paretic; Non-Par, non-paretic; NA, not available; –, data not relevant to the participant; Th, thalamus; IC, internal capsule; BG, basal ganglia; F, frontal; P, parietal; O, occipital; T, temporal; T-P, tempo-parietal; I, insula*.

**Table 2:** Participant demographic information.

	**Participants with stroke**	**Controls**
Gender	2 F/13 M	7 F/3 M
Dominant Arm	13 R/2 L/1 R+L	10 R/0 L/0 R+L
Age (μ ± σ (range))	59 ± 10 (43–75)	61 ± 7 (47–70)
Paretic Arm	8 R/7 L	–
Years since Stroke (μ ± σ (range))	10 ± 6 (3–28)	–

*F, female; M, male; R, right; L, left; μ, mean; σ, standard deviation; –, data not relevant to the participant*.

### 3.1. Motor Impairments

Participant strength in each arm during elbow flexion and extension, as well as UE FMA scores, are reported in Table 1. The strength of controls was nearly symmetric in each arm in elbow flexion [μ ± σ (range) MVTRatio: 0.96 ± 0.06 (0.88−1.05)] and extension [0.95 ± 0.06 (0.86−1.08)]. The strength of participants with stroke tended to be greater in their non-paretic arm than their paretic arm in elbow flexion [0.64 ± 0.24 (0.18−0.97)] and extension [0.70 ± 0.28 (0.25−1.21)]. The severity of motor impairments for the participants with stroke, according to their UE FMA score, spanned from mild to severe [μ ± σ (range) UE FMA: 37 ± 15 (12−62)].

For Stroke 7 and 12, the MVT of their paretic arm was less than 25% of the MVT of their non-paretic arm. As such, these participants would not be able to generate enough torque with their paretic arm to match 25% of the MVT of their non-paretic arm. To ensure that these participants could match the torque generated by their non-paretic arm, we set the target torques for Stroke 7 and 12 to 15% and 20% of the MVT of their reference arm, respectively.

### 3.2. Matching of Torques in Flexion

**Figure 3:**
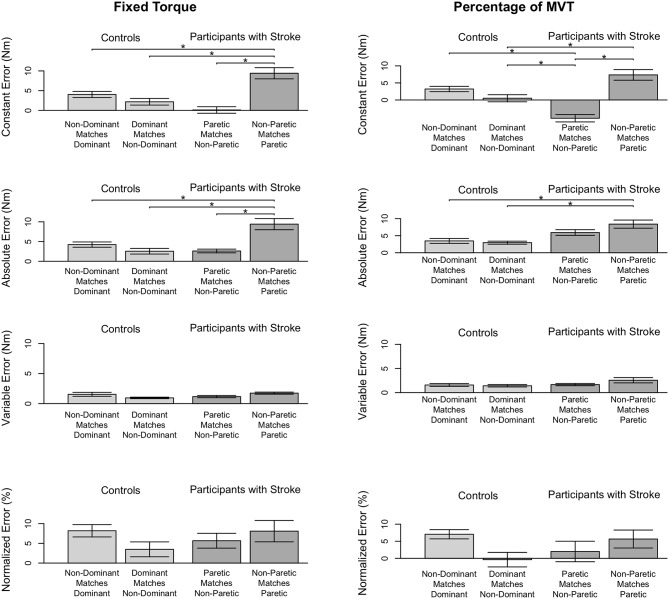
Participant accuracy and precision in matching torques in flexion. Mean (bar height) and standard error (error bars) of participants' constant errors, absolute errors, variable errors, and mean normalized errors when referencing each arm to match a **(Left)** fixed torque and **(Right)** percentage of their MVT. Significant differences are indicated by solid black horizontal lines with a star above.

Stroke 9 was unable to successfully match torques in elbow flexion due to an inability to hold the reference torque for 3 s. Therefore, this participant's data were not included in the following analyses. The accuracy and precision with which the remaining participants matched torques between arms in flexion as a function of their reference arm are summarized in Table 3 and Figure 3.

**Table 3:** Statistical results in flexion.

**Target**	**Group**	**Arm**	**μ_torque_**	**CE**	**AE**	**VE**
**torque**		**referenced**	**(Nm)**	**(Nm)**	**(Nm)**	**(Nm)**
	Controls	Dominant	5.2 ± 0.2	4.0 ± 2.4	4.2 ± 2.1	1.5 ± 1.0
Fixed		Non-dominant	5.2 ± 0.2	2.2 ± 2.6	2.5 ± 2.3	0.9 ± 0.4
	Participants	Non-paretic	5.3 ± 0.3	0.1 ± 3.1	2.6 ± 1.8	1.2 ± 0.7
	with Stroke	Paretic	4.8 ± 0.3	9.4 ± 5.3	9.4 ± 5.3	1.7 ± 0.6
	Controls	Dominant	13.6 ± 6.6	3.2 ± 2.5	3.5 ± 2.3	1.6 ± 0.9
Percentage		Non-dominant	13.2 ± 6.3	0.5 ± 3.3	3.0 ± 1.4	1.4 ± 0.8
	Participants	Non-paretic	14.4 ± 5.0	–5.4 ± 4.0	5.9 ± 3.1	1.7 ± 0.9
	with Stroke	Paretic	8.7 ± 4.9	7.4 ± 5.8	8.4 ± 4.5	2.6 ± 2.0

*Mean ± standard deviation are reported for every data entry. μ_torque_, mean magnitude of reference torque*.

#### 3.2.1. Fixed Torque Task

First, we address whether the errors when matching torques in flexion differed depending on the arm that participants referenced for the fixed torque task (Figure 3, Left). Constant error was affected by the arm that participants referenced (*p* < 0.001). Participants with stroke had a greater constant error when referencing their paretic arm than when referencing their non-paretic arm (*p* < 0.001) and than controls when referencing their non-dominant arm (*p* = 0.002) and dominant arm (*p* = 0.028). Absolute error was affected by the arm that participants referenced (*p* < 0.001). Participants with stroke had a greater absolute error when referencing their paretic arm than when referencing their non-paretic arm (*p* < 0.001) and than controls when referencing their non-dominant arm (*p* = 0.001) and dominant arm (*p* = 0.017). Variable error was affected by the arm that participants referenced (*p* = 0.035). However, *post-hoc* comparisons did not reveal any significant differences (*p* > 0.050).

Next, we indicate the results for the normalized errors when participants matched torques in flexion during the fixed torque task (Figure 3, Left, Bottom). The mean normalized error did not significantly differ depending on the arm that participants referenced (*p* > 0.050).

#### 3.2.2. Percentage of MVT Task

First, we address whether the errors when matching torques in flexion differed depending on the arm that participants referenced during the percentage of MVT task (Figure 3, Right). Constant error was affected by the arm that participants referenced (*p* < 0.001). Participants with stroke had a greater constant error when referencing their paretic arm than when referencing their non-paretic arm (*p* < 0.001) and than controls when referencing their non-dominant arm (*p* = 0.013). Also, participants with stroke when referencing their non-paretic arm had a constant error that was less than controls when referencing their non-dominant arm (*p* = 0.043) and dominant arm (*p* = 0.001). Absolute error was affected by the arm that participants referenced (*p* = 0.004). Participants with stroke had a greater absolute error when referencing their paretic arm than controls when referencing their non-dominant arm (*p* = 0.022) and dominant arm (*p* = 0.022). Variable error was not significantly affected by the arm that participants referenced (*p*> 0.050).

Next, we indicate the results for the normalized errors when participants matched torques in flexion during the percentage of MVT task (Figure 3, Right, Bottom). The mean normalized error did not significantly differ depending on the arm that participants referenced (*p*> 0.050).

### 3.3. Matching of Torques in Extension

#### 3.3.1. Fixed Torque Task

**Figure 4:**
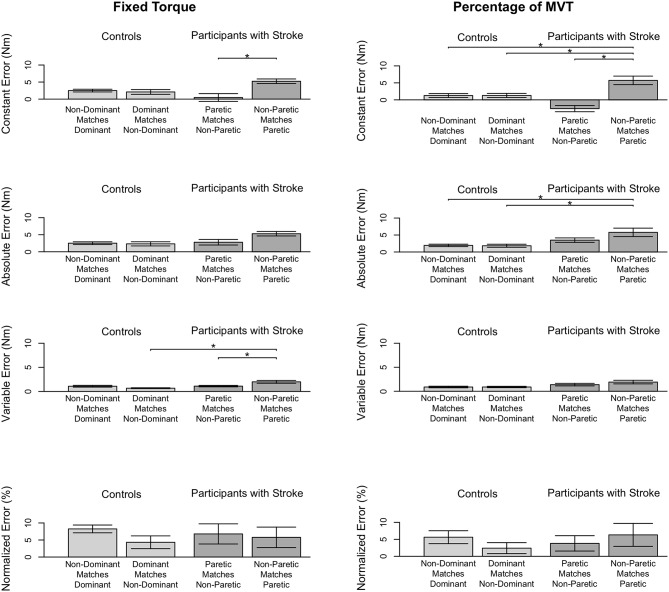
Participant accuracy and precision in matching torques in extension. Mean (bar height) and standard error (error bars) of participants' constant errors, absolute errors, variable errors, and mean normalized errors when referencing each arm to match a **(Left)** fixed torque and **(Right)** percentage of their MVT. Significant differences are indicated by solid black horizontal lines with a star above.

Stroke 10, 13, and 14 were unable to successfully match the fixed torque in elbow extension due to an inability to hold the reference torque for 3 s. Therefore, these participants' data were not included in the following analyses. The accuracy and precision with which the remaining participants matched torques between arms in extension as a function of their reference arm are summarized in Table 4 (Top) and Figure 4 (Left).

**Table 4:** Statistical results in extension. Mean±standard deviation are reported for every data entry. μ_torque_, mean magnitude of reference torque.

**Target**	**Group**	**Arm**	**μ_torque_**	**CE**	**AE**	**VE**
**torque**		**referenced**	**(Nm)**	**(Nm)**	**(Nm)**	**(Nm)**
	Controls	Dominant	5.2 ± 0.3	2.5 ± 1.3	2.5 ± 1.3	1.1 ± 0.6
Fixed		Non-dominant	5.1 ± 0.3	2.1 ± 2.2	2.3 ± 1.9	0.7 ± 0.3
	Participants	Non-paretic	5.2 ± 0.4	0.5 ± 4.0	2.8 ± 2.8	1.1 ± 0.5
	with Stroke	Paretic	4.8 ± 0.4	5.2 ± 2.3	5.3 ± 2.3	2.0 ± 1.0
	Controls	Dominant	9.2 ± 4.5	1.3 ± 1.8	1.9 ± 1.1	0.9 ± 0.5
Percentage		Non-dominant	8.9 ± 4.3	1.3 ± 1.9	1.8 ± 1.4	0.9 ± 0.5
	Participants	Non-paretic	11.4 ± 3.9	–2.5 ± 3.1	3.5 ± 2.3	1.4 ± 0.9
	with Stroke	Paretic	7.5 ± 3.6	5.7 ± 4.4	5.8 ± 4.3	1.9 ± 1.3

First, we address whether the errors when matching torques in extension differed depending on the arm that participants referenced during the fixed torque task. Constant error was affected by the arm that participants referenced (*p* = 0.005). Participants with stroke had a greater constant error when referencing their paretic arm than when referencing their non-paretic arm (*p* = 0.004). Absolute error was affected by the arm that participants referenced (*p* = 0.013). However, *post-hoc* comparisons did not reveal any significant differences (*p*> 0.050). Variable error was affected by the arm that participants referenced (*p* = 0.003). Participants with stroke had a greater variable error when referencing their paretic arm than when referencing their non-paretic arm (*p* = 0.021) and than controls when referencing their non-dominant arm (*p* = 0.003).

Next, we indicate the results for the normalized errors when participants matched torques in extension during the fixed torque task (Figure 4, Left, Bottom). The mean normalized error did not significantly differ depending on the arm that participants referenced (*p*> 0.050).

#### 3.3.2. Percentage of MVT Task

Stroke 10 and 13 were unable to successfully match torques in elbow extension due to an inability to hold the reference torque for 3 s. Therefore, these participants' data were not included in the following analyses. The accuracy and precision with which the remaining participants matched torques between arms in extension as a function of their reference arm are summarized in Table 4 (Bottom) and Figure 3 (Right).

First, we address whether the errors when matching torques in extension differed depending on the arm that participants referenced during the percentage of MVT task. Constant error was affected by the arm that participants referenced (*p* < 0.001). Participants with stroke had a greater constant error when referencing their paretic arm than when referencing their non-paretic arm (*p* < 0.001) and than controls when referencing their non-dominant arm (*p* = 0.050) and dominant arm (*p* = 0.050). Absolute error was affected by the arm that participants referenced (*p* = 0.021). Participants with stroke had a greater absolute error when referencing their paretic arm than controls when referencing their non-dominant arm (*p* = 0.049) and dominant arm (*p* = 0.007). Variable error was affected by the arm that participants referenced (*p* = 0.041). However, *post-hoc* comparisons did not reveal any significant differences (*p*> 0.050).

Next, we indicate the results for the normalized errors when participants matched torques in extension during the percentage of MVT task (Figure 3, Right, Bottom). The mean normalized error did not significantly differ depending on the arm that participants referenced (*p*> 0.050).

## 4. Discussion

The main findings of this work are that the participants with hemiparetic stroke: (i) had magnitudes of error similar to controls when referencing their non-paretic arm; (ii) had magnitudes of error greater than controls when referencing their paretic arm; and (iii) had similar results to controls, regardless of the arm referenced, when the torque that they generated at each arm was normalized by its corresponding MVT.

### 4.1. Torque-Matching Results

To determine whether individuals with hemiparetic stroke accurately matched torques on our task, we first needed to identify how accurately and precisely a group of individuals without neurological impairments matched torques between arms. Therefore, we first discuss the results of our controls, and then we discuss the results of our participants with stroke.

#### 4.1.1. Controls

We assessed whether our right-hand dominant controls matched torques differently depending on their arm dominance by quantifying their accuracy and precision in matching torques between arms when referencing each arm. Our results did not find controls to differ in their accuracy and precision in matching torques between arms depending on the arm referenced (i.e., dominant vs. non-dominant; *p* > 0.050). We acknowledge that a significant effect may have been obtained had a larger number of controls been tested. However, we point out that the effect size would likely have been relatively small, at least when compared to the degree of error observed in our participants with stroke. We cannot indicate whether accuracy and precision in matching torques would be affected by arm dominance in left-hand dominant individuals since we only tested right-hand dominant controls.

#### 4.1.2. Participants With Stroke

To begin, our results did corroborate the findings of other researchers when our participants with stroke referenced their paretic arm (4, 7, 8, 11). That is, our participants with hemiparetic stroke could not accurately match torques that they generated about their non-paretic elbow to those they generated about their paretic elbow. The accuracy of our participants with hemiparetic stroke in matching torques between arms when referencing their paretic arm was worse than when referencing their non-paretic arm, and than controls when referencing their non-dominant arm and dominant arm (*p* < 0.050). For the fixed torque task, the mean magnitude of error (i.e., absolute error) for our participants with stroke when referencing their paretic arm was 9.4 Nm in flexion and 5.3 Nm in extension. This magnitude of error was two to three times greater when participants with stroke referenced their paretic arm than when they referenced their non-paretic arm and than when controls referenced their dominant arm and non-dominant arm (see Tables 3, 4). Furthermore, Stroke 13, who was classified according to the rNSA as having absent elbow kinaesthesia and pressure sense in the paretic arm (see Table 1), matched torques between arms when referencing their paretic arm with an accuracy that was much greater than our controls. When referencing their paretic arm, this participant's mean magnitude of error on the fixed torque task in flexion was 16.4 Nm. Stroke 13 was not able to control their torques during the extension task so these results are not reported. We also highlight that the average torque-matching errors for our participants with stroke reached a 7:1 difference. Intuitively, this difference implies that a mass of 1 kg in the paretic arm is perceived as equivalent to a mass of 7 kg in the non-paretic arm. Therefore, our findings indicate that the errors in accurately judging the torques that one generates between arms can be substantial in individuals with hemiparetic stroke.

On the other hand, the magnitude of errors of our participants with hemiparetic stroke in matching torques between arms, when referencing their non-paretic arm, did not differ from the magnitude of errors of our controls when referencing their non-dominant arm and dominant arm (*p* > 0.050). For the fixed torque task, the mean magnitude of torque-matching error (i.e., absolute error) when participants with stroke referenced their non-paretic arm and controls referenced their dominant and non-dominant arm was 2.6, 4.2, and 2.5 Nm, respectively, in flexion and 2.8, 2.5, and 2.3 Nm, respectively, in extension. We also highlight that Stroke 13, who was classified according to the rNSA as having absent elbow kinaesthesia and pressure sense in the paretic arm (see Table 1), could match torques between arms when referencing their non-paretic arm with an accuracy that was similar to our controls. When referencing their non-paretic arm, this participant's mean magnitude of error in matching torques in flexion was 1.6 Nm. This finding that individuals with hemiparetic stroke can match torques between arms with a similar accuracy as individuals without neurological impairments is in contrast to what is known in the existing literature (4, 7, 8, 11). Even so, our finding that accuracy in matching depends on whether the non-paretic arm vs. paretic arm is referenced has been supported by other groups. Yen and Li (11) found that the accuracy in matching torques differed depending on whether individuals with hemiparetic stroke referenced their non-paretic arm vs. paretic arm. Likewise, Hirayama et al. (59) showed that the accuracy in locating one's thumb in space differed depending on whether individuals with lesions referenced their non-paretic arm vs. paretic arm.

As discussed above, the magnitude of errors (i.e., absolute error) did not significantly differ between the participants with stroke, when their paretic arm matched their non-paretic arm, and controls. That is, our participants with stroke, when referencing their non-paretic arm, matched torques with a similar degree of accuracy as our controls. Even so, our participants with stroke tended to undershoot the target torque when referencing their non-paretic arm and overshoot when referencing their paretic arm, whereas our controls, regardless of which arm they referenced, tended to overshoot to a lesser degree than the participants with stroke. Hence, while the magnitude of errors was similar, the torques generated by each arm differed in our participants with stroke and controls, as can be seen by the constant error.

Analyses based on the mean normalized errors did not reveal any differences regardless of the arm that participants with stroke and controls referenced (*p* > 0.050). Moreover, Stroke 13, who was classified according to the rNSA as having absent elbow kinaesthesia and pressure sense in the paretic arm (see Table 1), had nearly the same mean normalized error when referencing their paretic arm (15.3%) and non-paretic arm (15.7%) for the fixed torque task in flexion. Therefore, while the magnitude of errors differed depending on the arm referenced, the analyses that were run to investigate whether participants may match based on their perceived effort did not reveal a difference.

We recognize that our tested participants with stroke are heterogeneous in terms of lesioned hemisphere/location/size and arm dominance. We inspected our results according to these parameters, and we did not observe trends depending on any of these variables. Therefore, our findings indicate that, regardless of lesioned hemisphere/location/size and arm dominance, accuracy in matching torques was more severely affected when our participants with hemiparetic stroke referenced their paretic arm than their non-paretic arm.

### 4.2. Explaining the Asymmetry in Accurately Matching Torques

The most probable explanation to address why accuracy in matching torques between arms depends on whether individuals with hemiparetic stroke reference their paretic vs. non-paretic arm is aligned with the literature. Specifically, our findings support the notion that individuals with hemiparetic stroke match torques primarily based on their perceived effort. Post-hemiparetic stroke, an individual's paretic arm is weaker than their non-paretic arm. By rearranging the right-hand side of Equation (1), we can see that if the participant were to match the effort required to generate a torque at their non-paretic arm, ${\hat{\tau }}_{\text{non}-\text{paretic},i}$, to their paretic arm, ${\hat{\tau }}_{\text{paretic},i}$, then the matching torque would correspond to ${\hat{\tau }}_{\text{non}-\text{paretic},i}\;\sim \;\left(\frac{1}{\text{MVTRatio}}\right)\cdot {\hat{\tau }}_{\text{paretic},i}$. Applying the same rationale, if the participant were to match the effort required to generate a torque at their paretic arm, ${\hat{\tau }}_{\text{paretic},i}$, to their non-paretic arm, ${\hat{\tau }}_{\text{non}-\text{paretic},i}$, then the matching torque would be less, corresponding to ${\hat{\tau }}_{\text{paretic},i}\;\sim \text{ MVTRatio}\cdot {\hat{\tau }}_{\text{non}-\text{paretic},i}$. Typically, $\frac{1}{\text{MVTRatio}}>1$ and $\frac{1}{\text{MVTRatio}}<1$ in individuals with hemiparetic stroke. Hence, this explanation that our participants with hemiparetic stroke matched torques based on their perceived effort is reasonable for indicating why the magnitude of errors were greater and positive when their paretic arm was referenced, and smaller and negative when their non-paretic arm was referenced. We also provide an explanation to indicate why the mean normalized error was almost always greater than zero for each participant and arm referenced. We determined MVTs during a unimanual task and accuracy in matching torques during a bimanual task. Prior research has demonstrated that MVTs can differ depending on whether the task is unimanual vs. bimanual (60). Therefore, the mean normalized errors may have been closer to zero had we quantified MVTs during a bimanual task.

Additionally, we provide our original hypothesis to explain why we believed that individuals post-hemiparetic stroke inaccurately judge the torques that they generate between arms since this new hypothesis cannot be ruled out. Even so, we find the explanation that our participants with stroke were matching torques based on their perceived effort more probable. To begin, we point out that joint torque generation between the arms of a healthy adult is normally symmetric (10). However, post-hemiparetic stroke, joint torque generation becomes asymmetric (4, 7, 8, 10, 11, 61). Since the paretic arm lacks the control and strength of the non-paretic arm, during bimanual tasks individuals with hemiparetic stroke almost exclusively use their paretic arm to assist their non-paretic arm, not vice versa (62). Given this asymmetry when executing bimanual tasks, we hypothesized that individuals with hemiparetic stroke never learn to perceive their non-paretic arm in relation to their paretic arm (63). Consequently, their perception is only affected when using their non-paretic arm to assist their paretic arm to execute bimanual tasks. Given the motor control limitations occurring post-stroke in their paretic arm, we also hypothesized that these individuals learn to rely more heavily on their non-paretic arm than their paretic arm to perform activities of daily living. As such, they utilize their paretic arm only to the extent that is needed to achieve a similar degree of accuracy as prior to the stroke, resulting in their paretic arm undershooting the target torque.

### 4.3. Study Limitations

Limitations of this work include that participants were assessed on a single-degree-of-freedom isometric task, which is potentially an unfamiliar task. We chose this task since we wanted to: (i) avoid assessing participants on a multi-joint coordination task, which may give results that are more difficult to interpret, and (ii) isolate errors in judging torques from errors in judging movements, which may occur during an isotonic task. Future work could determine the ability of individuals with stroke to match torques between arms on multi-joint tasks and isotonic tasks when positional cues are included.

Another limitation of this study is that our results are summarized for a range of MVTs spanning between 4 and 40% at the paretic elbow. The literature provides conflicting evidence as to whether our findings will extend to MVTs beyond 40% (4, 19, 64). We underscore that a challenge faced in assessing larger percentages of MVT is fatigue, especially in individuals with stroke.

An additional limitation of this work is that our results are based on a sample size of fifteen individuals with stroke who have different levels of motor impairments. Future work could strengthen our results by identifying a quicker, and likely less precise assessment approach that would make it feasible to quantify the accuracy with which individuals can match torques between arms in a much larger group of participants.

### 4.4. Clinical Relevance

Rehabilitation practice focuses on movement limitations without considering how accurately an individual perceives the torques that they generate. Based on our results, we propose that the ability to accurately judge the torques that one generates may be important to study since it may hinder the ability of individuals with stroke to safely deal with unfamiliar or potentially dangerous bimanual tasks, such as carrying a platter with hot drinks, pushing a grocery cart, and caring for an infant. Perceiving that the torques generated in the non-paretic arm are as much as seven times greater than the torques generated in the paretic arm may contribute to the challenges that individuals with stroke face in controlling their upper limb movements during bimanual tasks, particularly when the task is novel.

Our results also support the notion that the erroneous judgment corresponds to how weak the paretic arm is in relation to the non-paretic arm. Consequently, the findings corroborate the idea that a large number of the 50–75% of stroke survivors who have hemiparesis (6, 65) inaccurately judge their self-generated torques, particularly when referencing their paretic arm during bimanual tasks.

Given the magnitude of the errors we observed, we propose that training individuals with hemiparetic stroke to more accurately perceive the torques that they generate may enable them to more successfully execute bimanual activities of daily living and avoid potentially dangerous situations. Strength training of the paretic arm could be one approach to reduce the extent of the erroneous judgment. However, this approach may not be very effective due to the losses in motor neural resources individuals post-hemiparetic stroke have that are necessary to drive their paretic upper limb (45–47). Based on the understanding that judgment errors are more evident when individuals post-hemiparetic stroke reference their paretic arm than their non-paretic arm, we propose that training these individuals to coordinate movements while focusing on their paretic arm may make them more aware and capable of rehabilitating their erroneous judgment. This proposed training protocol is novel when considering bimanual training approaches that have been developed to-date for individuals post-stroke [e.g., (5, 66–68)]. Future work can test the neuroplasticity of the erroneous judgment by determining whether training individuals with hemiparetic stroke to perceive their non-paretic arm in relation to their paretic arm during bimanual coordination tasks mitigates the degree of the errors. To conclude, we propose that an improved ability to accurately perceive the torques that one generates between arms may lead to greater usage of the paretic arm, resulting in survivors of a stroke gaining confidence and ability to engage in daily activities.

## Ethics Statement

This study was carried out in accordance with the recommendations of the Northwestern University Institutional Review Board with written informed consent from all subjects. All subjects gave written informed consent in accordance with the Declaration of Helsinki. The protocol was approved by the Northwestern University Institutional Review Board.

## Author Contributions

NG, NvdH, JMD, and JPD: study design, data interpretation, and manuscript editing. NG, NvdH, and JMD: data acquisition. NG and NvdH: data analysis. NG: manuscript preparation.

### Conflict of Interest Statement

The authors declare that the research was conducted in the absence of any commercial or financial relationships that could be construed as a potential conflict of interest. The reviewer AS declared a shared affiliation, with no collaboration, with the authors to the handling editor at time of review.
